# Isolated right ventricular löffler endocarditis secondary to allergic bronchopulmonary aspergillosis

**DOI:** 10.1093/ehjcr/ytag003

**Published:** 2026-01-08

**Authors:** Rikako Horie, Yasuhisa Nakao, Kenta Horie, Osamu Yamaguchi

**Affiliations:** Department of Cardiology, Pulmonology, Nephrology, and Hypertension, Ehime University Graduate School of Medicine, Toon, Ehime 791-0295, Japan; Department of Cardiology, Pulmonology, Nephrology, and Hypertension, Ehime University Graduate School of Medicine, Toon, Ehime 791-0295, Japan; Department of Hematology, Clinical Immunology, and Infectious Diseases, Ehime University Graduate School of Medicine, Toon, Ehime 791-0295, Japan; Department of Cardiology, Pulmonology, Nephrology, and Hypertension, Ehime University Graduate School of Medicine, Toon, Ehime 791-0295, Japan; Medical Genome Center, National Cerebral and Cardiovascular Center, Suita, Osaka 564-8565, Japan

**Keywords:** Löffler endocarditis, Allergic bronchopulmonary aspergillosis, Eosinophilia, Right ventricular thrombosis

A 74-year-old man with a history of chronic productive cough presented with rapidly progressive bilateral leg oedema over one month. Transthoracic echocardiography showed preserved left ventricular systolic function, enlargement of the right atrium and ventricle (*Panel A*), reduced right ventricular wall motion, and an isoechoic mass at the right ventricular apex (yellow arrowheads in *Panel B*). Cardiac computed tomography confirmed an apical thrombus (white arrowheads in *Panel C*), and cardiac magnetic resonance imaging demonstrated delayed enhancement of the right ventricular free wall, predominantly in the endocardial to mid-myocardial layers (red arrowheads in *Panel D*). Laboratory tests revealed persistent eosinophilia (1000–1500/μL) over the previous five years, and bone marrow examination excluded idiopathic hypereosinophilic syndrome. Chest computed tomography showed a progressive right lower-lobe infiltrate (*Panel E*, black circle), and bronchoscopy revealed a mucus plug. Aspergillus-specific IgG and IgE antibodies were positive, establishing allergic bronchopulmonary aspergillosis (ABPA) as the underlying cause of secondary eosinophilia. Anticoagulation and corticosteroid therapy led to marked thrombus reduction, normalization of eosinophil counts, and resolution of respiratory and heart failure symptoms.

**Figure:**
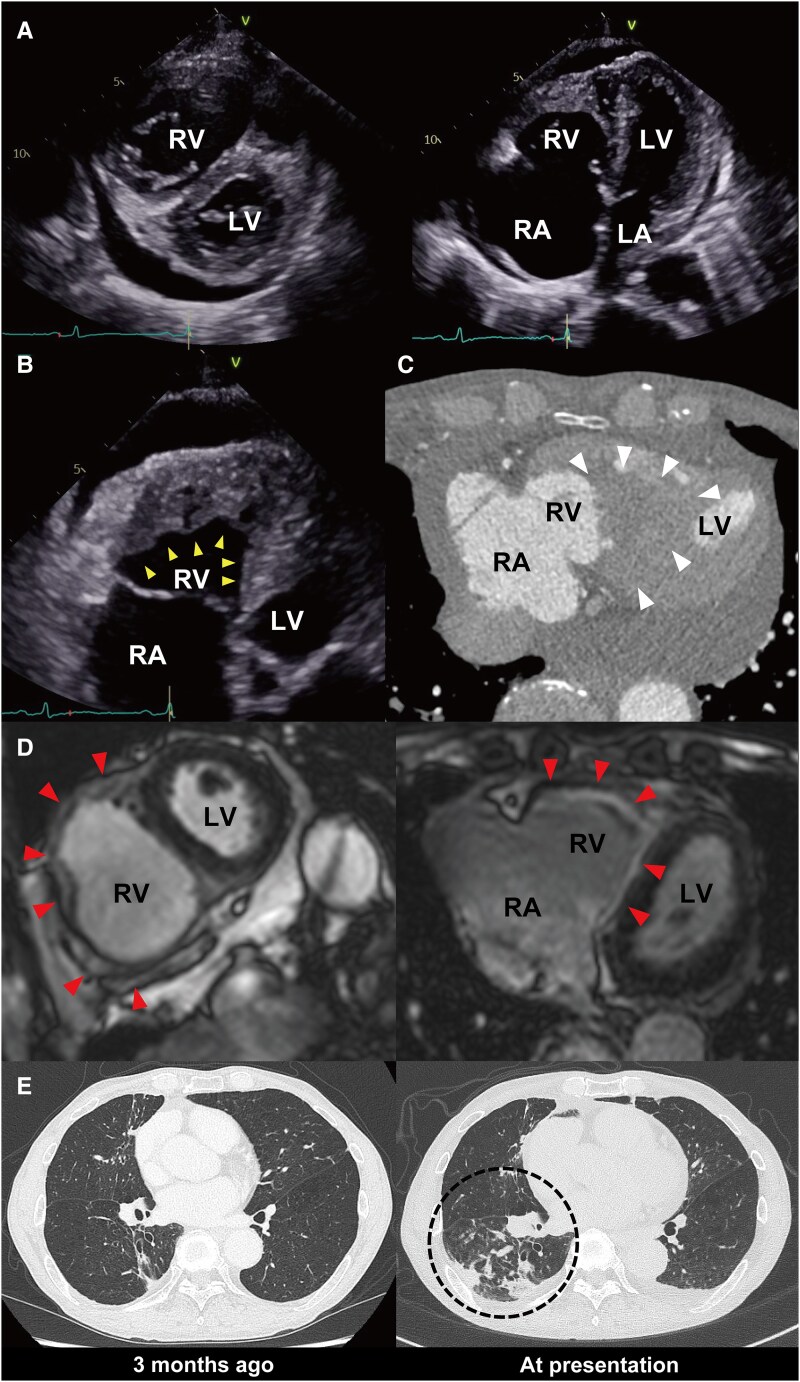


This case highlights that ABPA-related eosinophilia can cause Löffler endocarditis confined to the right ventricle. In contrast to idiopathic hypereosinophilic syndrome—typically associated with aggressive inflammation, biventricular myocardial damage, and extensive intracardiac thrombi—secondary eosinophilia may progress more slowly and be diagnosed while still limited to the right ventricle.^1–3^ Clinicians should maintain a high index of suspicion for intracardiac thrombus in patients with ABPA, as early identification and appropriate therapy may prevent life-threatening cardiac complications.

## Data Availability

The data underlying this article cannot be shared publicly due to patient privacy concerns. The data are available from the corresponding author upon reasonable request.
